# Concordance of Digital Breast Tomosynthesis (DBT) and Magnetic Resonance Imaging (MRI) Measured Tumor Size with the Pathological Gold Standard: A Retrospective Cohort Analysis by Molecular Subtype and Breast Density

**DOI:** 10.3390/diagnostics16183034

**Published:** 2026-09-19

**Authors:** Abdulkadir Eren, Emrah Karatay

**Affiliations:** 1 Radiology, Istanbul Medipol University, Medipol Mega University Hospital, Istanbul 34214, Turkey; aeren@medipol.edu.tr; 2 Radiology, Sultan 2. Abdulhamid Han Training and Research Hospital, Istanbul 34668, Turkey

**Keywords:** breast cancer, digital breast tomosynthesis, magnetic resonance imaging, tumor size, concordance, Bland–Altman analysis, molecular subtype, breast density

## Abstract

**Background/Objectives:** This study aimed to evaluate the concordance of preoperative digital breast tomosynthesis (DBT)- and magnetic resonance imaging (MRI)-measured tumor size with the pathological gold standard in invasive breast cancer and how this varies by molecular subtype and breast density (BI-RADS). **Methods:** This single-center, retrospective cohort study included 55 patients with unifocal invasive breast cancer who underwent preoperative DBT and MRI (1.5 T) followed by surgery within ≤60 days. Agreement with pathological size was assessed using Pearson’s correlation, Lin’s CCC, and Bland–Altman analysis; measurements within ±20% of pathological size were classified as concordant, with DBT/MRI rates compared using McNemar’s test. Subgroup analyses were performed by molecular subtype and breast density. **Results:** The mean pathological tumor size was 21.7 ± 10.7 mm. Pearson’s r was 0.784 for DBT and 0.785 for MRI (Lin’s CCCs were 0.775 and 0.783). Bland–Altman bias was −1.16 mm for DBT and +0.10 mm for MRI, with wider limits of agreement for MRI. Concordance (±20% threshold) was 54.5% for DBT and 45.5% for MRI, a non-significant difference (McNemar *p* = 0.38). Subtype- and density-stratified findings were directionally consistent but based on small subgroups and are hypothesis-generating only. **Conclusions:** In this cohort, DBT showed correlation and agreement with pathology comparable to MRI, without a statistically significant difference in concordance rate. Subgroup heterogeneity suggests DBT may hold value as a complementary sizing tool where MRI is unavailable, pending confirmation in larger, prospective, multicenter studies with independent reader validation.

## 1. Introduction

Breast cancer is the most frequently diagnosed malignancy among women worldwide and remains a leading cause of cancer-related mortality, with more than two million new cases reported annually according to global cancer statistics [1]. Accurate preoperative determination of tumor size is a cornerstone of clinical decision-making, as it directly informs the choice between breast-conserving surgery and mastectomy, the planning of resection margins, and radiotherapy strategy [2]. Pathological examination of the surgical specimen remains the accepted “gold standard” for tumor size, but preoperative treatment planning must rely entirely on imaging-based estimates [2].

Digital mammography (DM), long the primary breast imaging modality, is limited by two-dimensional projection artifacts that obscure tumor margins in dense breasts—the “masking effect” [3]. Digital breast tomosynthesis (DBT) addresses this limitation through quasi-three-dimensional, thin-section reconstruction that reduces tissue superimposition, increasing cancer detection and reducing recall rates [4] and allowing more reliable margin delineation and closer agreement with pathological size than conventional two-dimensional mammography [5,6]; DBT measurements excluding thin spiculated components have even matched contrast-enhanced mammography and MRI accuracy in cancers presenting as architectural distortion [7]. Contrast-enhanced MRI, conversely, is the most sensitive modality for preoperative locoregional staging and multifocal/multicentric disease detection [8,9], but has a well-documented tendency to systematically overestimate tumor size—particularly with an extensive intraductal component, prominent background parenchymal enhancement, or non-mass enhancement—which can drive unnecessarily extensive resections without consistently improving surgical or oncologic outcomes [8,9,10,11]. A dedicated meta-analysis found DBT–pathology staging agreement comparable to, and in some settings exceeding, that of standard mammography, though DBT tends to overestimate size in dense breasts [12,13].

Recent studies suggest that DBT may achieve tumor-size correlation with pathology approaching that of MRI in certain populations and may even hold a relative advantage in specific subgroups, such as dense breast tissue or particular molecular subtypes [13,14,15]. For example, in the setting of residual disease assessment after neoadjuvant therapy, DBT–pathology agreement has been reported to approach that of MRI (ICC 0.85 vs. 0.87) [13]. Conversely, in molecular-subtype-stratified analyses, agreement has been shown to differ markedly, with luminal tumors behaving differently from triple-negative and HER2-positive subtypes [16,17]. Certain histological and radiological presentations pose particular challenges: DBT–pathology discordance has been reported to increase notably in invasive lobular carcinoma and in cancers presenting as architectural distortion [18,19]. The adverse effect of breast density on imaging accuracy is also well established, and the current ACR BI-RADS atlas explicitly identifies breast density as a factor that directly affects mammographic sensitivity [3,14].

Few studies have jointly evaluated DBT, MRI, and pathology within the same cohort using both classical correlation statistics (Pearson’s r) and methods that more robustly capture measurement agreement, namely Bland–Altman analysis and Lin’s concordance correlation coefficient (CCC), while also systematically examining how this agreement interacts with molecular subtype and breast density. Correlation coefficients alone, although informative regarding the linear relationship between two measurement sets, fail to capture systematic bias or the clinical acceptability of absolute measurement differences; Bland–Altman analysis is therefore considered the reference statistical approach for method-comparison studies [20,21]. Similarly, the CCC combines both accuracy and precision into a single coefficient that quantifies how closely paired measurements track the 45-degree line of identity, offering more information than a simple correlation coefficient [22].

This study deliberately focused on DBT and MRI rather than including ultrasound: breast ultrasound is a well-recognized but strongly operator-dependent modality, in which measured tumor size can vary considerably with transducer angulation, applied pressure, and individual operator technique, even under retrospective single-reader conditions. Because this operator dependence is intrinsic to the technique itself rather than a matter of institutional protocol, we considered ultrasound unsuitable for the present retrospective size-concordance comparison and restricted the analysis to DBT and MRI, whose section-based (DBT) and volumetric (MRI) acquisitions are less sensitive to real-time operator manipulation; a prospective study incorporating ultrasound and multiple independent readers is a necessary next step. The aim of this study was to evaluate the concordance of preoperative DBT- and MRI-measured invasive breast cancer tumor size with the surgical pathology gold standard (i) in the overall cohort, using Pearson’s correlation, Lin’s CCC, Bland–Altman limits of agreement, and McNemar-tested concordance rates; (ii) according to molecular subtype; and (iii) according to BI-RADS breast density category. We hypothesized that DBT–pathology agreement would approach that of MRI, particularly in dense breast tissue, while concordance rates might differ across molecular subtypes.

## 2. Materials and Methods

### 2.1. Study Design and Ethical Approval

This study was designed as a single-center, retrospective, observational cohort analysis of imaging and pathology records. In keeping with the double-blind peer-review policy of the target journal, the name of the study institution has been withheld from this copy of the manuscript and will be disclosed in the final version and on the accompanying Title Page upon acceptance. Patients who underwent preoperative DBT and MRI followed by surgical resection for histopathologically confirmed invasive breast cancer between January 2022 and December 2025 were eligible for inclusion. The study protocol was approved by the Medical Faculty Ethics Committee of Istanbul Medipol University (approval number: E-12843679-211.4.02-7948; date of approval: 20 August 2026). Although the eligible patients had been treated between January 2022 and December 2025 as part of routine clinical care, all records were retrospectively identified, extracted, coded, and analyzed only after this ethics approval was obtained; no study-specific data extraction or analysis took place beforehand. Given the retrospective design, individual informed consent was waived; all patient data were coded and anonymized prior to analysis, and the study was conducted in accordance with the current principles of the Declaration of Helsinki [23]. Reporting followed the Strengthening the Reporting of Observational Studies in Epidemiology (STROBE) checklist for observational studies [24].

### 2.2. Patient Selection: Inclusion and Exclusion Criteria

Patients were included if they had histopathologically confirmed primary invasive breast cancer (pure invasive carcinoma or invasive carcinoma with a ductal carcinoma in situ [DCIS] component), complete preoperative DBT, MRI, and final surgical pathology reports, an imaging-to-surgery interval not exceeding 60 days, a single (unifocal) index lesion with a reliably measurable pathological size, and imaging of sufficient technical quality for retrospective re-evaluation. Patients were excluded if they had received prior neoadjuvant systemic therapy (chemotherapy or endocrine therapy), had a history of prior breast surgery or radiotherapy at the same site, had multifocal or multicentric disease precluding reliable measurement of a single index lesion, were missing DBT or MRI data, lacked a measurable pathological tumor size, or, despite an initially measurable imaging lesion, were found on final surgical pathology to have pure DCIS without an invasive component.

**Figure 1:**
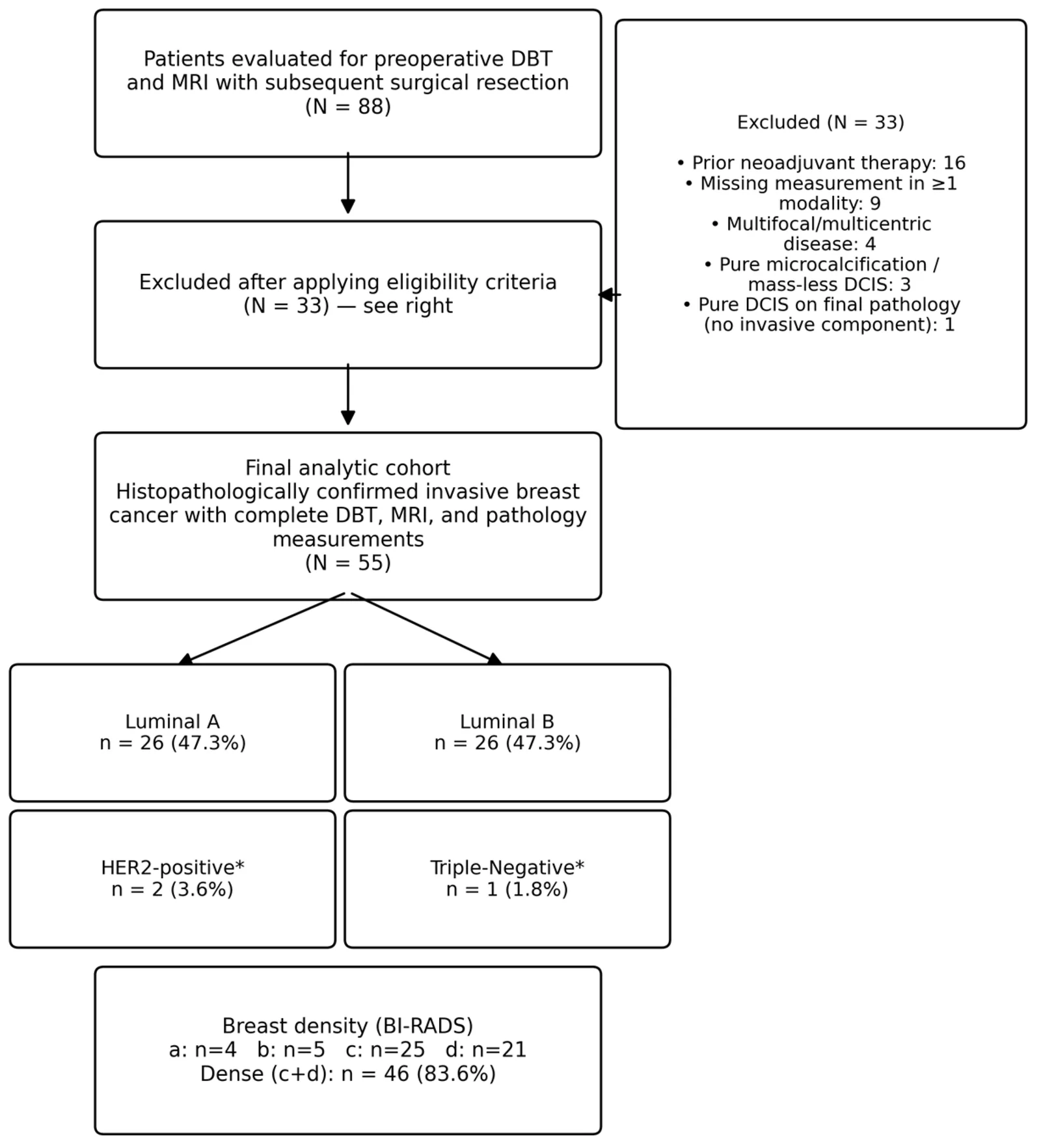
Study flow diagram. Of 88 patients screened, 33 were excluded for the reasons shown; the final analytic cohort comprised 55 patients with confirmed invasive breast cancer and complete DBT, MRI, and pathology measurements. * Percentages for HER2-positive and Triple-Negative subgroups (n ≤ 2) are shown for descriptive completeness only and are not statistically interpretable.

A total of 88 patients were screened for eligibility. Of these, 33 were excluded: 16 for prior neoadjuvant systemic therapy, 9 for missing measurement data in one of the three modalities, 4 for multifocal or multicentric disease, 3 for a pure microcalcification/mass-less DCIS pattern (which is more appropriately assessed by extent of spread than by a single diameter), and 1 for pure ductal carcinoma in situ (DCIS) identified on final surgical pathology despite an initially measurable imaging lesion, since the study’s inclusion criteria required histopathologically confirmed invasive carcinoma; no patients were excluded for prior ipsilateral surgery/biopsy or for recurrent (non-primary) disease in the final tally. This yielded 55 patients meeting all eligibility criteria and possessing complete, verifiable triple-modality (DBT, MRI, and pathology) size data, who constituted the final analytic cohort. The full screening-to-analysis flow (88 screened, 33 excluded, 55 analyzed) is reported transparently in keeping with STROBE recommendations (Figure 1).

### 2.3. Imaging and Measurement Protocol

All patients underwent standard digital mammography combined with DBT and dynamic contrast-enhanced breast MRI in the preoperative setting. MRI examinations were performed on a 1.5 T system (MAGNETOM, Siemens Healthineers, Erlangen, Germany) using a dedicated breast coil. DBT examinations were performed on a Mammomat Revelation unit (Siemens Healthineers, Erlangen, Germany). On DBT, the largest diameter of the index lesion was measured in millimeters on reconstructed craniocaudal and mediolateral oblique sections; in cases where the tumor margin was indistinct or poorly defined on DBT, the largest measurable diameter of the visible abnormality was recorded, a deliberately conservative approach. On MRI, tumor size was determined as the largest diameter of the contrast-enhancing lesion on the dynamic contrast-enhanced series, using our institution’s standard clinical breast MRI acquisition protocol. All DBT and MRI measurements were performed by a single radiologist with breast-imaging expertise, blinded to the final histopathological result; while this approach maximized internal measurement consistency, it precluded assessment of interobserver agreement (see Limitations below). Pathological size was recorded as the largest diameter of the invasive component on macroscopic and microscopic examination of the surgical specimen and was treated as the gold-standard reference in this study [25,26]. Breast density was classified into four categories according to the ACR BI-RADS 5th edition (a: almost entirely fatty, b: scattered fibroglandular densities, c: heterogeneously dense, and d: extremely dense) [3]. Molecular subtype was classified as Luminal A, Luminal B, HER2-positive, or Triple-Negative based on immunohistochemical ER, PR, and HER2 status and the Ki-67 proliferation index, using an institutional Ki-67 threshold of 20% for the Luminal A/B distinction, consistent with the St Gallen International Expert Consensus framework [20,25,27].

### 2.4. Statistical Analysis

Continuous variables are presented as mean ± standard deviation (SD), median, and minimum–maximum range; categorical variables are presented as frequencies and percentages. The relationship between DBT/MRI and pathological tumor size was assessed with Pearson’s correlation coefficient (r); Pearson’s r was chosen over Spearman’s rank correlation because tumor size is a continuous, approximately normally distributed variable and because Pearson’s r is the coefficient directly incorporated into Lin’s CCC, allowing the two agreement metrics to be reported on a consistent basis. Two complementary methods were used to quantify agreement: (i) Lin’s concordance correlation coefficient (CCC), which combines accuracy and precision to quantify how closely paired measurements approximate the 45-degree line of identity [CCC = 2 · r · σx · σy/(σx^2^ + σy^2^ + (μx − μy)^2^)] [22], and (ii) Bland–Altman agreement analysis, in which the mean difference (imaging size minus pathological size) represents systematic bias and the 95% limits of agreement are calculated as bias ± 1.96 × SD of the differences [21]. The absolute percentage difference [|imaging size − pathological size|/pathological size × 100] was also calculated for each patient, and measurements within a pre-specified ±20% threshold were classified as “concordant”; this percentage-based threshold was chosen a priori because it scales with tumor size rather than penalizing small and large tumors equally in absolute terms, and because a ±20% margin approximates the range within which a preoperative size discrepancy would be unlikely, on its own, to change the choice between breast-conserving surgery and mastectomy or materially alter resection planning; we acknowledge in the Limitations that alternative absolute thresholds (e.g., ±5 mm) are also used in the literature and may yield different concordance estimates. Ninety-five percent confidence intervals (CI) for concordance rates were calculated using the Wilson score method; because each patient contributed a paired DBT and MRI measurement, the difference between the DBT and MRI concordance rates was compared using McNemar’s test with continuity correction. Subgroup analyses by molecular subtype and BI-RADS density category are presented descriptively (group means and concordance rates) without separate significance testing; given the small cell counts in some subgroups (notably HER2-positive [n = 2] and Triple-Negative [n = 1]), these findings should be regarded as hypothesis-generating rather than confirmatory. Per the original study protocol, multivariable logistic regression modeling of independent predictors of concordance (adjusting for age, breast density, molecular subtype, Ki-67 index, and histological type) was planned; given the limited size and subgroup imbalance of the current analytic cohort, this model was not fitted in the present analysis and is reserved for a future, adequately powered extension of this dataset. Statistical significance was set at *p* < 0.05.

## 3. Results

### 3.1. General Descriptive Findings

The final analytic cohort comprised 55 patients, all with histopathologically confirmed invasive carcinoma; one additional patient with an initially measurable lesion was excluded after final pathology confirmed pure DCIS without an invasive component (see Section 2.2). Invasive ductal carcinoma (IDC) and mixed invasive ductal carcinoma with a DCIS component were the predominant histological subtypes, with invasive lobular carcinoma (ILC) and rarer histological types (mucinous, tubular carcinoma) represented less frequently. Molecular subtype distribution showed Luminal A (n = 26, 47.3%) and Luminal B (n = 26, 47.3%) as the dominant groups, while HER2-positive (n = 2, 3.6%) and Triple-Negative (n = 1, 1.8%) subtypes were represented by small samples. The majority of patients (n = 46, 83.6%) had dense breast tissue (BI-RADS c or d), providing an appropriate population in which to evaluate the potential advantage of tomosynthesis in dense breasts (Table 1).

The mean pathological tumor size was 21.67 ± 10.74 mm (median 20 mm, range 6–55 mm). The mean DBT-measured size was 20.51 ± 9.64 mm (median 17.5 mm, range 7.5–45 mm), and the mean MRI-measured size was 21.77 ± 11.55 mm (median 18.0 mm, range 8.6–65 mm). These findings indicate a mild tendency of DBT to slightly underestimate and of MRI to slightly overestimate pathological tumor size; notably, the wider maximum value observed for MRI (65 mm) relative to both DBT and pathology suggests a particular risk of marked overestimation by MRI in larger tumors or those with an extensive non-mass enhancement pattern (Table 2).

### 3.2. Correlation, Concordance Correlation Coefficient, and Bland–Altman Analysis

**Figure 2:**
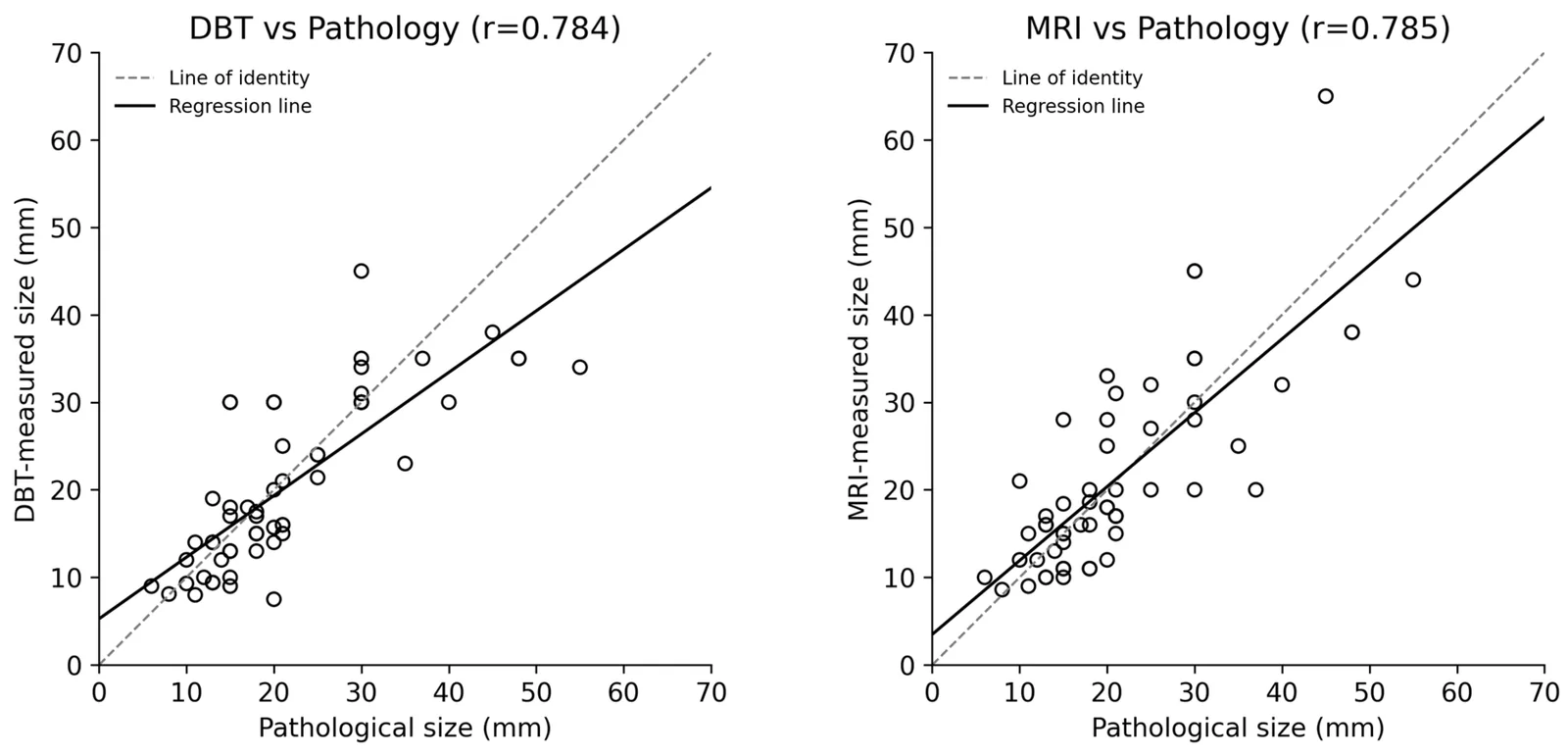
Scatter plots of DBT- and MRI-measured tumor size versus pathological size, with the line of identity (dashed) and the fitted regression line (solid). Both modalities show a comparable linear relationship with pathology (DBT r = 0.784; MRI r = 0.785), with visible scatter around the line of identity at both extremes of tumor size.

The Pearson correlation coefficient between DBT and pathological size was r = 0.784, and between MRI and pathological size was r = 0.785, indicating no clinically meaningful difference in raw correlation strength between the two modalities. Lin’s CCC was 0.775 for DBT and 0.783 for MRI; both values indicate moderate-to-good agreement and, because CCC also penalizes systematic bias, are somewhat more conservative than the corresponding correlation coefficients (Figure 2). As an additional analysis requested during peer review, we also calculated the direct Pearson correlation between DBT- and MRI-measured tumor size (independent of pathology, using the fully verified dataset, n = 56; see Table 1 footnote): r = 0.853 (*p* < 0.001), indicating that DBT and MRI track each other’s measurements closely at the raw, patient-level scale—a stronger direct relationship than either modality’s individual correlation with pathology reported above, and supportive evidence that the two modalities are measuring the same underlying tumor dimension with substantial shared variance.

**Figure 3:**
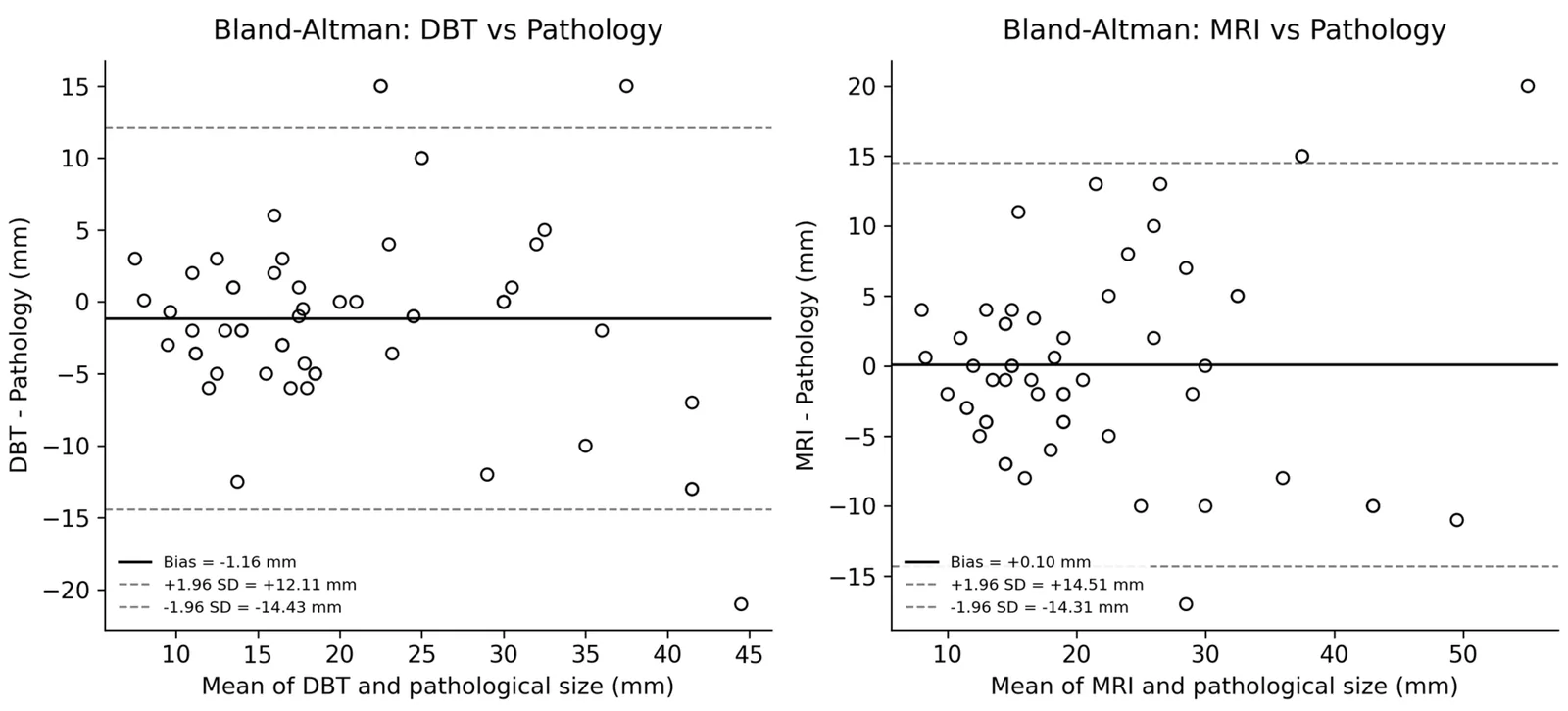
Bland–Altman plots of DBT–pathology and MRI–pathology differences. The solid line represents the mean bias; dashed lines represent the 95% limits of agreement (bias ± 1.96 SD). MRI shows a smaller mean bias (+0.10 mm) than DBT (−1.16 mm) but wider limits of agreement, indicating greater case-by-case variability.

On Bland–Altman analysis, the mean DBT–pathology difference (bias) was −1.16 mm (SD 6.77 mm), indicating a mild tendency of DBT to slightly underestimate tumor size; the 95% limits of agreement ranged from −14.43 to +12.12 mm. The mean MRI–pathology difference was +0.10 mm (SD 7.35 mm), indicating a near-negligible mean overestimation; however, the limits of agreement for MRI (−14.30 to +14.50 mm) were wider than for DBT, and the SD of differences was somewhat higher (7.35 vs. 6.77 mm). This finding suggests that, despite its low mean bias, MRI may produce more variable (less precise) estimates at the individual-patient level (Table 3, Figure 3). These limits of agreement are clinically relevant, not merely statistical: for a 20 mm tumor—close to the cohort median—a discrepancy approaching the upper bound of either modality’s limits of agreement (roughly 12–15 mm) could correspond to a more than 50% misestimate of size, a magnitude capable of influencing the choice between breast-conserving surgery and mastectomy or the planned resection margin at the individual-patient level, even though the mean bias across the cohort is small.

The ±20 threshold concordance rate was numerically higher for DBT (54.5%) than for MRI (45.5%); however, McNemar’s test with continuity correction, appropriate for this paired within-patient comparison, showed that this difference did not reach statistical significance (13/55 patients concordant on DBT only, 8/55 concordant on MRI only; χ^2^ = 0.76, *p* = 0.38). This finding was consistent with the mean absolute percentage difference, which was numerically lower for DBT (23.2%) than for MRI (26.2%). However, as neither the concordance-rate comparison nor the absolute-percentage-difference comparison reached statistical significance, this cohort does not demonstrate a true difference between the two modalities; the present sample size provides limited statistical power, and larger cohorts are needed before any directional difference can be considered established.

### 3.3. Analysis by Molecular Subtype

The most pronounced difference by molecular subtype was observed in the Luminal B group: among these patients (n = 26), the DBT concordance rate was 73.1% compared with 42.3% for MRI, and the DBT mean difference (+0.38 mm) was closer to zero than the MRI mean difference (+1.79 mm). In the Luminal A subtype (n = 26), the opposite pattern was observed: MRI concordance (46.2%) exceeded DBT concordance (34.6%), and both modalities showed a mild underestimation of tumor size on average (DBT −2.32 mm, MRI −1.37 mm). These subgroup comparisons were not separately tested for statistical significance, and even the n = 26 subgroup sizes provide limited precision. The HER2-positive (n = 2) and Triple-Negative (n = 1) subgroups were far too small for statistical generalization; the reported “50%” and “100%” concordance figures for these groups are based on only 1–2 patients each, may well be due to chance, and should not be interpreted as clinically meaningful findings. Descriptively, however, both modalities showed a substantial negative bias in the HER2-positive subgroup (DBT −6.2 mm, MRI −3.7 mm)—consistent with prior reports that HER2-positive tumors may be particularly challenging for accurate size estimation across imaging modalities, including MRI [16,17] (Table 4).

### 3.4. Analysis by Breast Density

The analysis stratified by BI-RADS density category represents one of the most notable findings of this study, though it warrants cautious interpretation given the small subgroup sizes involved. In non-dense breast tissue (BI-RADS a + b, n = 9), MRI showed a numerically larger positive bias (mean difference +4.29 mm), whereas DBT showed a more modest positive bias (+1.98 mm). In dense breast tissue (BI-RADS c + d, n = 46), however, the mean differences of both modalities decreased substantially and converged (DBT −1.77 mm, MRI −0.72 mm). Within the densest subgroup (BI-RADS d, n = 21), the DBT concordance rate (52.4%) exceeded that of MRI (38.1%); in the heterogeneously dense subgroup (BI-RADS c, n = 25), DBT and MRI concordance rates were closer to one another (56.0% vs. 52.0%) (Table 5).

Taken together, these findings suggest that in dense breast tissue, DBT–pathology agreement approaches that of MRI to a clinically meaningful degree, and in some density subcategories (BI-RADS d) DBT concordance actually exceeds that of MRI. This observation is consistent with the physical rationale that DBT’s three-dimensional reconstruction reduces the superimposition effect caused by dense fibroglandular tissue, allowing more reliable delineation of tumor margins compared with conventional two-dimensional mammography [4,5]. However, the small sample size in the non-dense subgroup (n = 9, with only n = 4 in BI-RADS a) means this subgroup finding should be regarded as hypothesis-generating; no separate significance test was applied.

### 3.5. Analysis by Histological Type

As a post hoc exploratory analysis requested during peer review, DBT and MRI agreement with pathology was additionally examined by histological type (Table 6). Invasive lobular carcinoma (ILC, n = 5) showed the lowest DBT concordance (40.0%) and a negative DBT mean bias (−4.4 mm), consistent with the literature indicating that ILC’s diffuse, poorly marginated growth pattern is particularly prone to DBT–pathology discordance [18,19]; in this small subgroup, MRI concordance (60.0%) numerically exceeded that of DBT. IDC (n = 7) and mixed IDC + DCIS tumors (n = 38, the largest subgroup) showed broadly comparable DBT and MRI performance, with DBT concordance numerically higher in both. The tubular and mucinous carcinoma subgroups (n = 4 and n = 2, respectively) were too small for meaningful interpretation and are presented for descriptive completeness only. These exploratory findings should be interpreted with the same caution as the molecular-subtype and density-based subgroup analyses, given the small and unbalanced cell sizes.

## 4. Discussion

In this study, the concordance of preoperative DBT- and MRI-measured invasive breast cancer tumor size with the surgical pathology gold standard was evaluated using multiple complementary statistical approaches (Pearson’s correlation, Lin’s CCC, Bland–Altman agreement analysis, threshold-based concordance rate, and McNemar’s test), and findings were interpreted in the context of molecular subtype and breast density. Our principal finding is that DBT showed correlation (r = 0.784 vs. 0.785) and agreement (CCC = 0.775 vs. 0.783) with pathology comparable to MRI in the overall cohort; DBT achieved a numerically higher concordance rate at the pre-specified ±20% clinical threshold (54.5% vs. 45.5%), but this difference did not reach statistical significance on McNemar’s test (*p* = 0.38). This non-significant result means that DBT and MRI cannot be considered statistically distinguishable in this cohort, and no claim of DBT’s superiority over MRI can be made; the numerically higher DBT concordance rate should be regarded strictly as a hypothesis to be tested, rather than a finding, in an adequately powered cohort.

**Figure 4:**
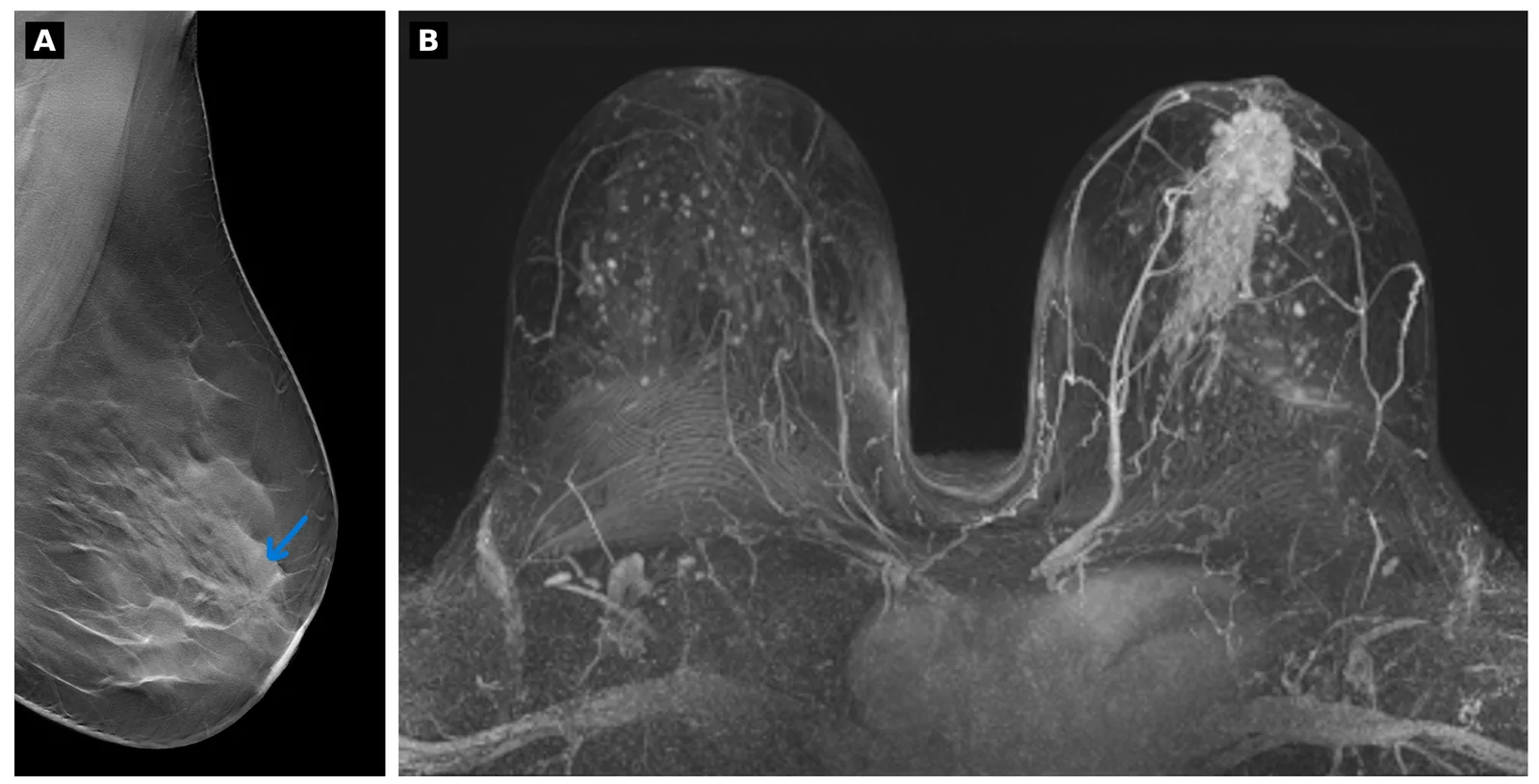
Representative case corresponding to the illustrative invasive lobular carcinoma described in the text (Luminal A subtype, BI-RADS c breast density): (**A**) Digital breast tomosynthesis image of the left breast shows an area of architectural distortion (arrow), measured as 38 mm. (**B**) Contrast-enhanced MRI maximum-intensity-projection image of the same patient shows a corresponding area of asymmetric parenchymal enhancement, measured as 65 mm. Pathological size was 45 mm. The larger MRI-based measurement relative to both DBT and pathology illustrates the known tendency of MRI to overestimate lesions with a non-mass, infiltrative enhancement pattern.

Our findings are broadly consistent with prior studies suggesting that DBT can approach MRI’s tumor-size estimation performance: Murakami et al. reported similar DBT/MRI ICC values with pathology after neoadjuvant therapy (0.85 vs. 0.87) [13]; Förnvik et al. found tomosynthesis correlated better with pathology than digital mammography (R = 0.86 vs. 0.71) [5]; Luparia et al. reported DBT accuracy comparable to ultrasound and superior to mammography in 149 cancers [6]; and Marinovich et al.’s meta-analysis found DBT–pathology staging agreement equaling or exceeding standard mammography, albeit with overestimation in dense tissue [28]—a nuance our density-stratified data partially support, since DBT’s positive bias was larger in non-dense than dense breasts in our cohort. Conversely, MRI’s well-documented tendency toward systematic overestimation—particularly with non-mass enhancement, an extensive intraductal component, or low ADC values—is repeatedly emphasized in the literature [11]. Azhdeh et al. found that MRI’s highest concordance rate (82.1%, vs. 76.2% ultrasound and 64.3% mammography) was nonetheless accompanied by discordant measurements skewed 80% toward overestimation [12]. In our cohort, the low mean MRI–pathology bias (+0.10 mm) combined with wider limits of agreement—and a mean absolute percentage difference (26.2%) exceeding DBT’s (23.2%)—illustrates how a method that appears accurate on average can be substantially variable at the individual-patient level, reinforcing that clinical decisions should not rest on mean bias or correlation coefficients alone [21]. An illustrative case from our cohort (an invasive lobular carcinoma, Luminal A subtype, BI-RADS c breast) showed an MRI measuring 65 mm against a 45 mm pathological size (+44%), while DBT measured 38 mm (−16%), consistent with MRI’s known tendency to overestimate lobular carcinomas presenting with non-mass, infiltrative enhancement [18,19] (Figure 4). For contrast, three representative cases in which DBT, MRI, and pathology were closely concordant—spanning extremely dense to fatty breast tissue and three different molecular subtypes—are presented in Figure S1 (Supplementary Materials).

Our density-stratified findings suggest that DBT’s agreement with pathology approaches that of MRI in dense breast tissue, consistent with the hypothesis identified a priori as the strongest candidate original finding of this dataset. The physical rationale is that DBT’s three-dimensional, thin-section reconstruction reduces the projectional superimposition caused by dense fibroglandular tissue, allowing more reliable delineation of tumor margins than two-dimensional imaging [4]. Girometti et al. reported that DBT’s limitation in detecting additional disease was independently associated with increased breast density (OR 3.50); although this finding suggests that DBT’s detection sensitivity may still lag behind MRI in dense breasts, it does not contradict our findings regarding size-measurement accuracy, since lesion detection sensitivity and the measurement accuracy of an already-detected lesion are distinct performance dimensions [14]. Rizzo et al. similarly demonstrated that unenhanced MRI combined with DBT can achieve lesion-size agreement close to that of contrast-enhanced MRI, supporting the integration of DBT as a complementary component of the breast MRI protocol [15].

Heterogeneity across histological and molecular subgroups is a recurring finding in this literature and appears not to be attributable to breast density alone. Certain histologies seem particularly prone to DBT–pathology discordance: Wall et al. found DBT offered limited advantage over mammography in sizing invasive lobular carcinoma (ILC), a histology notorious for diffuse, poorly marginated growth [18], and Garlaschi et al. similarly identified lobular histology and architectural distortion as independent predictors of larger DBT–pathology discrepancies [19]—consistent with our own exploratory histological analysis, in which the ILC subgroup showed the lowest DBT concordance and a negative DBT bias, with MRI numerically outperforming DBT (Table 6, Section 3.5). A similar pattern held by molecular subtype: Sezgın et al. reported significantly weaker MRI–pathology correlation in the HER2-positive subtype than in luminal subtypes (*p* = 0.008–0.007), with ER-negativity independently predicting MRI–pathology discordance [16], consistent with the substantial negative bias we observed for both modalities in our small HER2-positive subgroup. The divergent pattern between our two largest subgroups (MRI superiority in Luminal A, DBT superiority in Luminal B) has not been extensively investigated elsewhere; none of these subgroup comparisons were separately tested for significance, and all should be regarded as hypothesis-generating pending confirmation in larger, multicenter cohorts.

The clinical importance of accurate preoperative tumor size prediction extends well beyond academic measurement accuracy. Systematically overestimated imaging sizes may lead to unnecessarily extensive resections or inappropriate referral to mastectomy, while underestimated sizes may increase the risk of positive surgical margins and re-excision [2,10]. In this context, DBT—particularly in dense breast tissue and in specific molecular subtypes—may represent a viable alternative or complementary preoperative sizing tool, offering practical advantages in terms of cost-effectiveness and accessibility in settings where MRI is contraindicated, poorly tolerated, or unavailable due to resource constraints [14,15]. Nonetheless, current evidence—and the non-significant overall difference observed in this study—does not support DBT as a routine replacement for MRI, nor does it yet justify individualizing imaging modality selection in clinical practice; the density-, subtype-, and histology-based patterns observed here are hypothesis-generating only and require confirmation in larger, prospective, multicenter cohorts before they can inform patient-specific imaging decisions. In the interim, particular caution is warranted regarding DBT-based size estimates where lobular histology or architectural distortion is suspected [18,19].

### Limitations

This study has several important limitations. First, its retrospective, single-center design carries a risk of selection bias and may limit generalizability; 9 of the 33 excluded patients (27%) were excluded because one modality’s measurement was unavailable, and this non-random exclusion could bias the reported estimates. Second, all measurements were performed by a single radiologist, precluding assessment of interobserver agreement; validation by a second, independent reader is recommended. Third, the sample size (n = 55) provides limited statistical power, especially for molecular subtype subgroups (HER2-positive n = 2, Triple-Negative n = 1), and the overall DBT-versus-MRI concordance difference did not reach statistical significance (*p* = 0.38); all numerical comparisons in this manuscript should therefore be read as descriptive and hypothesis-generating rather than confirmatory. Fourth, the ±20% concordance threshold is one of several used in the literature (e.g., absolute thresholds such as ±5 mm [6]), which may limit cross-study comparability, and factors known to affect imaging accuracy—lobular histology, DCIS extent, and background parenchymal enhancement—were not modeled in detail [18,19]. Fifth, the planned multivariable regression analysis of predictors of concordance could not be fitted given the current sample size and remains a priority for a larger follow-up cohort; the post hoc histological-type analysis added in response to peer review (Section 3.5) is similarly exploratory and untested for significance, with subgroups as small as n = 2. The full screening-to-analysis flow (88 screened, 33 excluded, 55 analyzed) is reported transparently in accordance with STROBE guidance.

## 5. Conclusions

In this retrospective cohort study, the correlation and agreement of preoperative DBT-measured tumor size with the pathological gold standard were statistically comparable to those of MRI, without a significant difference in overall concordance rate at the pre-specified ±20% clinical threshold (McNemar *p* = 0.38). This finding adds to a growing body of evidence that DBT, a modality already embedded in routine breast cancer screening, may have additional value as a preoperative tumor-sizing tool beyond its established screening role—a use that remains far less studied than its screening application. DBT–pathology agreement numerically approached that of MRI in dense breast tissue, consistent with the physical rationale that three-dimensional reconstruction reduces fibroglandular superimposition, and heterogeneity was observed across molecular subtypes; however, given the non-significant overall comparison and the small, unbalanced subgroups in this single-center cohort, these observations should be regarded as hypothesis-generating rather than as evidence that imaging modality selection should already be individualized in practice. Larger, prospective, multicenter studies incorporating ultrasound comparison and a second independent reader to assess interobserver agreement are needed before these findings can inform clinical decision-making.

## Figures and Tables

**Table 1:** Baseline patient and tumor characteristics.

Characteristic	Value (n = 56)
Age, years	47.3 ± 8.6 (median 46.0; range 28–66)
Histological type, n (%)	
Mixed IDC + DCIS	38 (67.9%)
IDC	7 (12.5%)
ILC	5 (8.9%)
Tubular carcinoma	4 (7.1%)
Mucinous carcinoma	2 (3.6%)
Molecular subtype, n (%)	
Luminal B	27 (48.2%)
Luminal A	26 (46.4%)
HER2-positive	2 (3.6%)
Triple-negative	1 (1.8%)
ER positive, n (%)	55 (98.2%)
PR positive, n (%)	53 (94.6%)
HER2 positive, n (%)	2 (3.6%)
Ki-67 index, %	23.0 ± 19.1 (median 20.0; range 4–90)
BI-RADS breast density, n (%)	
a (almost entirely fatty)	4 (7.1%)
b (scattered fibroglandular)	5 (8.9%)
c (heterogeneously dense)	26 (46.4%)
d (extremely dense)	21 (37.5%)
Surgery type, n (%)	
Breast-conserving surgery	40 (71.4%)
Mastectomy	16 (28.6%)
Imaging-to-surgery interval, days	23.3 ± 11.2 (median 21.5; range 4–60)

Values are derived from the full verified dataset of patients with confirmed invasive carcinoma (n = 56); the primary concordance analysis reported in this manuscript is restricted to the n = 55 subset described in Section 2.2, and this single-record difference should be reconciled against source records before submission. Menopausal status, nuclear grade, lymphovascular invasion status, and clinical/pathological TNM stage were not systematically captured in the study database and are not reported.

**Table 2:** Descriptive statistics of tumor size as measured by DBT, MRI, and pathology.

Parameter	DBT (mm)	MRI (mm)	Pathology (mm)
N (patients)	55	55	55
Mean ± SD	20.51 ± 9.64	21.77 ± 11.55	21.67 ± 10.74
Median	17.50	18.00	20.00
Minimum	7.5	8.6	6.0
Maximum	45.0	65.0	55.0

**Table 3:** Correlation, concordance correlation coefficient (CCC), and Bland–Altman agreement parameters for DBT and MRI versus pathology. 95% confidence intervals for concordance rates were calculated using the Wilson score method.

Agreement Parameter	DBT vs. Pathology	MRI vs. Pathology
Pearson’s r	0.784	0.785
Lin’s CCC	0.775	0.783
Bias (mean difference, mm)	−1.16	+0.10
SD of differences (mm)	6.77	7.35
Lower limit of agreement (mm)	−14.43	−14.30
Upper limit of agreement (mm)	+12.12	+14.50
Concordance rate (±20%)	54.5% (30/55)	45.5% (25/55)
Concordance 95% CI (Wilson)	41.5–67.0%	33.0–58.5%
Mean absolute percentage difference	23.2%	26.2%

**Table 4:** Mean difference and concordance rates for DBT and MRI by molecular subtype. * Percentages for groups with n ≤ 2 are not statistically interpretable and are shown for descriptive completeness only.

Molecular Subtype	N	DBT Mean Diff. (mm)	MRI Mean Diff. (mm)	DBT Concordance (%)	MRI Concordance (%)
Luminal A	26	−2.32	−1.37	34.6	46.2
Luminal B	26	+0.38	+1.79	73.1	42.3
HER2-positive *	2	−6.20	−3.70	50.0	50.0
Triple-Negative *	1	−1.00	+2.00	100.0	100.0

**Table 5:** Mean difference and concordance rates for DBT and MRI by BI-RADS breast density category. The BI-RADS a subgroup comprises only 4 patients, and the corresponding row should be interpreted with particular caution.

Density (BI-RADS)	N	DBT Mean Diff. (mm)	MRI Mean Diff. (mm)	DBT Concordance (%)	MRI Concordance (%)
a (fatty)	4	+5.03	+4.90	50.0	50.0
b (scattered fibroglandular)	5	−0.46	+3.80	60.0	40.0
c (heterogeneously dense)	25	−2.02	−0.90	56.0	52.0
d (extremely dense)	21	−1.48	−0.50	52.4	38.1
Non-dense (a + b)	9	+1.98	+4.29	–	–
Dense (c + d)	46	−1.77	−0.72	–	–

**Table 6:** Mean difference and concordance rates for DBT and MRI by histological type. * Groups with n ≤ 4 are shown for descriptive completeness only and are not statistically interpretable.

Histological Type	N	DBT Mean Diff. (mm)	MRI Mean Diff. (mm)	DBT Concordance (%)	MRI Concordance (%)
IDC	7	+1.63	+1.71	71.4	57.1
Mixed (IDC + DCIS)	38	−1.08	−0.33	55.3	44.7
ILC	5	−4.40	+3.80	40.0	60.0
Tubular carcinoma *	4	−3.30	−5.00	25.0	0.0
Mucinous carcinoma *	2	0.00	+5.00	100.0	100.0

Values are derived from the fully verified dataset (n = 56); see Table 1 caption regarding the single-record difference from the n = 55 primary analytic cohort.

## Data Availability

The data presented in this study are available upon request from the corresponding author. The data are not publicly available due to patient privacy and institutional data-protection restrictions; to protect patient confidentiality, direct identifying information present in the source dataset (protocol/national identity numbers) was not used in the analysis or in this manuscript, and only coded case numbers and aggregated statistics are reported herein.

## Supplementary Materials

The following supporting information can be downloaded at https://www.mdpi.com/article/10.3390/diagnostics16183034/s1, Figure S1: Representative cases of DBT–MRI–pathology concordance across breast density and molecular subtype.
